# Engram formation in psychiatric disorders

**DOI:** 10.3389/fnins.2014.00118

**Published:** 2014-05-28

**Authors:** Peter J. Gebicke-Haerter

**Affiliations:** ^1^Medical Faculty Mannheim, Central Institute of Mental Health, Institute of Psychopharmacology, Heidelberg UniversityMannheim, Germany; ^2^Progrs. de Farmacología y Inmunología, Facultad de Medicina, Universidad de ChileSantiago, Chile

**Keywords:** neuronal networks, computational simulation, synaptic engram, cross-frequency coupling, gap junctions, post-translational modifications, epigenetics, schizophrenia

## Abstract

Environmental factors substantially influence beginning and progression of mental illness, reinforcing or reducing the consequences of genetic vulnerability. Often initiated by early traumatic events, “engrams” or memories are formed that may give rise to a slow and subtle progression of psychiatric disorders. The large delay between beginning and time of onset (diagnosis) may be explained by efficient compensatory mechanisms observed in brain metabolism that use optional pathways in highly redundant molecular interactions. To this end, research has to deal with mechanisms of learning and long-term memory formation, which involves (a) epigenetic changes, (b) altered neuronal activities, and (c) changes in neuron-glia communication. On the epigenetic level, apparently DNA-methylations are more stable than histone modifications, although both closely interact. Neuronal activities basically deliver digital information, which clearly can serve as basis for memory formation (LTP). However, research in this respect has long time neglected the importance of glia. They are more actively involved in the control of neuronal activities than thought before. They can both reinforce and inhibit neuronal activities by transducing neuronal information from frequency-encoded to amplitude and frequency-modulated calcium wave patterns spreading in the glial syncytium by use of gap junctions. In this way, they serve integrative functions. In conclusion, we are dealing with two concepts of encoding information that mutually control each other and synergize: a digital (neuronal) and a wave-like (glial) computing, forming neuron-glia functional units with inbuilt feedback loops to maintain balance of excitation and inhibition. To better understand mental illness, we have to gain more insight into the dynamics of adverse environmental impact on those cellular and molecular systems. This report summarizes existing knowledge and draws some outline about further research in molecular psychiatry.

## Introduction

A widely-accepted hypothesis of the origin of schizophrenia posits that perinatal insults play major roles as triggering events in the development of the disorder. One of these insults is reduced oxygen supply, a condition not uncommon at delivery and supposedly affecting the brain more than other organs. Evidently, this cannot be the whole story. Mental disorders like any other disease develop stepwise or, described in a more abstract way, as sequences of yes/no decisions or “bifurcations,” a term used in mathematics (chaos theory) for a long time. Along these lines, one could understand the development of an organism or the brain as the formation of a tree-like structure, unique and specific for each single individual, devolving and maintaining characteristic dynamics during the whole life span. That means, that the course of single events is not linear (50% yes/ 50% no decisions for each bifurcation are only a statistical probability, but do not reflect biological reality). Some may reinforce a certain direction, others may compensate for events drifting into adverse directions. Compensatory mechanisms inherent to all biological systems play a pivotal role, anyway. Dependent on the strength of adverse impacts and their specific accumulation over time, a disease may become evident and diagnosable within a short period of time or, as observed in most diseases of the brain, only after a long time of concealed progression. Consequently, this implies a sustained process of disease development divided in two parts, the “unnoticed” and the “recognizable” part. Clearly, both parts are relative, depending on the available technological tools to identify and confirm signs of a mental illness.

Obviously, this not only applies for the development of psychiatric disorders but also for the status of health, mental skills like intelligence and geniality. In order to understand these processes in more detail, we have to elaborate on the way how learning and long-term memory is produced and stored in the Central Nervous System (CNS), assuming that induction and progression of those disorders are based on the same mechanisms. The long-term memory process can be generally divided into four distinct stages: learning, consolidation, storage and retrieval. The storage of information is a delicate balancing act. The nervous system has to decide, which of the incoming new information is worth to be selected for consolidation and long-term storage, and which should be discarded, to avoid overload of storage capacity. Thereafter, stored informations have to be sufficiently stable, but have to be available for rapid retrieval, as well.

Several studies identified the hippocampus as an important site for consolidating labile short-term memories into more stable long-term memories. After hippocampal-dependent consolidation, these memories are thought to be transferred to and stored in the cortex (Wiltgen et al., 2004). This somewhat delayed consolidation process is believed to reflect the systems-level of memory consolidation, encompassing gradual reorganization of additional brain circuits and distribution of short-term memory to more remote cortical areas for permanent storage. For further consolidation, long-term memories in cortical areas seem to require so-called reactivation of the hippocampus that acts as a coincidence-regenerator (Squire et al., 2004). Hence, the hippocampus initially works with the neocortex to consolidate memory. Subsequently, changes in the neocortex over time become more essential for storing the information by restructuring connectivities among more distant cortical areas (Squire, 2009). This interaction is supposed to result in gradual strengthening of the cortical–cortical connectivity for permanent storage (Wittenberg and Tsien, 2002). Once those cortical connections become strongly consolidated, long-term memories can remain stable even in the absence of the hippocampus. In the literature those cortically stored long-term memories are often referred as remote memories (Wang et al., 2006).

Apparently any input, any contact with the environment impacts on the abovementioned yes/no decisions forming “engrams,” which are theoretical constructs of the most basic units of memory. The term was used first by Semon (1921), describing it as “the enduring though primarily latent modification in the irritable substance produced by stimulus.” In an extension of this view, the accumulation or synergistic combination of engrams may result in reinforced disease development or bolstered mental health, which entails the notion that any of these complex outcomes can only result from the interplay of many “memory traces.” This leads us to issues of the physical substrates of memory formation and its stabilization over long time—supposedly until the end of life of a human being.

## Search for physical substrates of engrams

### Neuronal networks and computational neuroscience

Already over 40 years ago, specific cells (neurons) of the prefrontal cortex were identified as putative “memory cells” by their sustained activities over extended periods of time upon a triggering stimulus (Fuster and Alexander, 1971). This stimulated the search for neuronal ensembles or sites of the brain encoding memory (Sakaguchi and Hayashi, 2012). The brain region most thoroughly studied in this respect is the hippocampus. At least short-term memory appears to be encoded by cells of this region and may be gradually redistributed to other sites in the brain and stored as long-term memory. Whether there is really a strict regional separation between different types of memory and whether long-term memory is identical to the information stored as short-term memory is still a matter of an ongoing debate (Sutherland and Lehmann, 2011). Another issue worth mentioning in this context is the temporal aspect, i.e., that re-experiencing exactly the same event some time later does not necessarily activate the same neurons and therefore may not result in an identical memory trace (Vazdarjanova and Guzowski, 2004). The high likelihood of these subtle variations stems from the marked combinatorial power that even resides in one given hippocampal granule cell with approximately 40,000 inputs. From those, roughly 400 are needed to trigger an action potential (McNaughton et al., 1981). A selection of any 400 combinations of inputs from the total of 40,000 results in a very large number (1.21 × 10^96^) of combinations. Facing this enormous combinatorial variability available to any neuron in the hippocampus, it appears logical that even identical signals arriving from the environment, but at different times, trigger distinct selections of input combinations in different single neurons, and eventually produce accumulating, distinct patches of long-lasting memory. Conversely, it may be assumed, that retrieval of a particular memory at different times does not recruit the same group of neurons. In consequence, there is a number of similar neuronal networks encoding memory traces of the same event received at different times resulting in the development of relational networks (Eichenbaum, 2004). Opposing this concept, it has been reported some time ago, that the same ensemble of neurons in the hippocampus of rats is activated upon the same stimulus displayed a second time 30 min later (Guzowski et al., 1999). The Arc cat-FISH method used in this study for showing neuronal activation, however, does not necessarily prove that the labeled neurons encode the memory.

**Figure 1:**
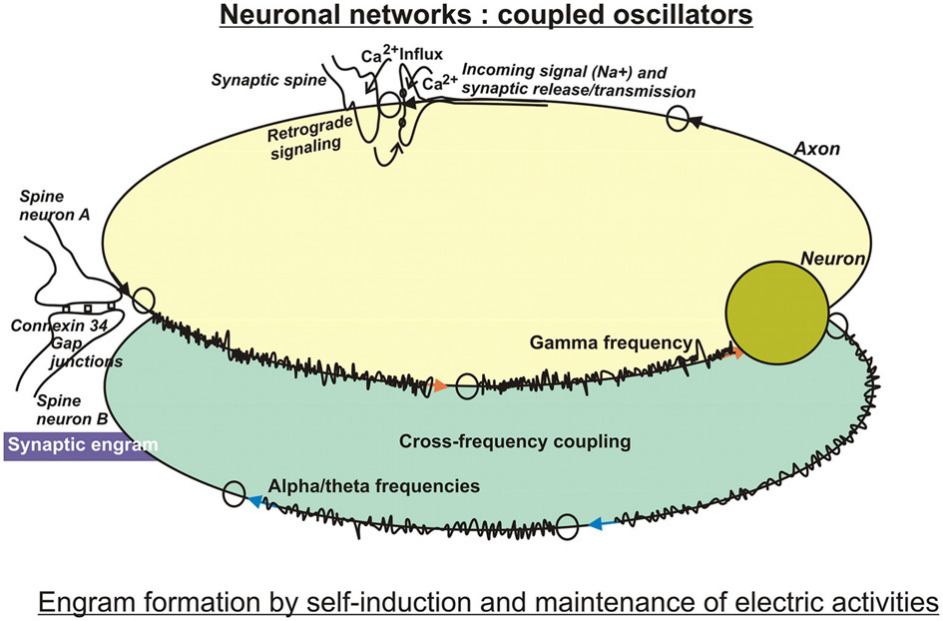
**In search for substrates of engrams**. Artificial neuronal networks have been studied intensively by computational tools, because they are reminiscent of integrated circuits used in computer technology. Basically, the biological neuronal networks encode incoming information in digital ways, because their firing depends on critical thresholds that control yes/no responses. Encoding may be performed by the frequencies or by the amplitudes of firing, and may be modulated by cross-frequency coupling of two or more interacting networks. These circuits are characterized by self-induction and maintenance of electric activities. Therefore, the computational approach entails storage and retrieval of information on the electro (-physiological) level using feedforward and feedback loops. By and large, these models have been modified by taking into account specific biological features, such as time-delay functions as observed at neurotransmission at synapses, and weighted inputs owing to different synaptic strengths, just to mention a few improvements (for more, see text). The so-called electrical synapses connected by gap junctions serve lateral spreading of information in dendritic trees, and gave rise to the notion of a “synaptic engram.” This special information processing entailed an additional challenge in models of computational neuroscience.

Along these lines, but apparently biased by the huge surge of computer technologies, the search for the physical basis of engrams, or how learning and memory are encoded, concentrated on neuronal ensembles or networks in analogy to integrated electrical circuits. In this respect, computational neurosciences have advanced considerably our understanding of the functioning and learning capacities of neuronal networks using mathematical tools, such as machine-learning algorithms (Hinton et al., 2006), or performing mathematical simulations with the goal to establish systems of artificial intelligence. Because they are dealing with networks in the sense of electronic circuits, they are essentially composed of “neurons” and their electric activities, albeit with modifications added upon increasing knowledge from biology. An attractive feature of neuronal circuitries appears to be their oscillatory behavior that may be useful to encode information derived from spatially nonhomogeneous and time-independent inputs into spatio-temporal codes. Hence, improvements of performance and robustness of the systems were achieved by coupling oscillators (two or more neuronal networks, Figure 1) and synchronize their frequencies (Orosz et al., 2009). Optionally, self-sustained feedback mechanisms resembling molecular oscillators, that are triggered by short-term memory, have been discussed along with memory consolidation (Bailey et al., 2004).

In this context, it was believed very early on, that a complete understanding of the wiring systems of neurons would enable us to obtain a detailed view into neuronal patterns of activity and resultant higher-level cognitive processes (Perkel, 1988). This “connectionism,” assuming that neuronal networks are comparable to logical circuit elements that follow simple threshold rules, was the most convenient approach to apply physical sciences in brain research. More recent efforts to obtain a complete “connectome” of the mammalian nervous system renewed the interest in “connectionism” (Lichtman et al., 2008). However, no neuronal network is hard-wired. For this reason, these promising efforts had to be improved by taking into account some additional important and typical features of biological neuronal networks: their functioning in highly nonlinear, dynamic ways (Ramirez et al., 2004) with complex and reciprocal excitation-inhibition and bursting activities (Koch et al., 2011). Additionally, detailed aspects of (synaptic) plasticity and resultant changes of activity patterns are hard to be reconciled with concepts of connectionism. Therefore, by and large, algorithms have been developed that included one or the other of those additional features.

For example, the introduction of a time-delay function (Zou et al., 2012), which is owed to the intrinsic properties of synaptic neurotransmission, appeared to improve the memory storage capacities of neural networks without substantially increasing their size. It turned out, that the time-lag in conjunction with the firing process at the synapse, combined with inhibitory feedback and with the absolute refractory period, features that were already contained in the well-known Hodgkin–Huxley model (Foss et al., 1996), could indeed result in basic types of oscillations, as mentioned above. To further reconcile the fast dynamics of neuronal activities with the relatively slow dynamics of synaptic weights (more or less frequent use of a synapse), the previously developed Adaptive Resonance Theory (ART) (Carpenter and Grossberg, 1987) helped to reach some state of machine learning and to increase the capacity for memory storage and retrieval in a network of two layers of neurons (or neuron populations).

A further aspect, conflicting with engram formation on computational grounds, is the peculiarity of neurons to show rapid leakage of currents out of their membranes, which makes them inherently “forgetful.” As a remedy, this leakage of currents was compensated for by insertion of memory cells in circuits containing positive-feedback loops. These cells are made to replace leaked currents exactly at the time when they are lost. Unfortunately, positive-feedback loops have the tendency to run out of control when they are not strictly balanced. Therefore, optional mechanisms with negative-derivative feedback were studied to evaluate their effects on generating sustained activity and temporal integration. Indeed, as published recently (Lim and Goldman, 2013), mechanisms based on negative-derivative feedback control maintain activity (memory) for long durations in a neural network after the stimulus has been removed, and may even serve as temporal integrators of their inputs.

Another conflicting aspect is synaptic plasticity, a process progressing throughout life, and characterized by axonal sprouting and formation of new axo-dendritic contacts (neuropoiesis). These new synaptic sprouts that have been observed along dendritic trees of layer V pyramidal cells, are formed in response to “experience” or “environmental cues” and are stimulated by electrophysiological events, like long term potentiation (LTP) in the proximal dendritic domains. Especially, the distinctions and the interactions between LTP and long-term memory are major issues of research. It has been shown that consolidation of memory can even be disrupted by new learning, but also by brain trauma, seizures, or by blockade of transcription or translation (Dudai, 2004). Moreover, disruption of stabilized memory traces may not only occur upon new learning, but also upon retrieval of stored information. Hence, memory retrieval is believed to induce a temporary period of lability that allows an existing association to be updated (Finnie and Nader, 2012). Consequently, the synapses storing the engram are destabilized, and undergo a period of deconstruction and protein degradation that makes the synapses malleable (Lee, 2010; Jarome et al., 2011). Nevertheless, also for these dynamic processes that undergo stabilization and destabilization, theoretical models of computation have been developed using fuzzy algorithms that result in about 39.7 × 10^12^ possible fuzzy engrams in the human cortex (Lopez et al., 2006). Obviously, this is just a theoretical number of all the options. In real biological systems, they are markedly reduced to a limited range of possibilities by optimizations. The initial phase of the learning process is triggered or can be reinforced by LTP and depression (LTD), or by other experience-dependent electrophysiological events increasing the efficiency of transmission (Bank and Schacher, 1992). The resultant structural and functional changes of synapses and postsynaptic responses gave rise to the exploration of additional learning algorithms, one of which is the synaptic weight association training (SWAT) algorithm (Wade et al., 2010). It is an improvement of the spike timing dependent plasticity (STDP) algorithm that was combined with Bienenstock–Cooper–Munro (BCM) theory to implement a learning rule (Benuskova and Kasabov, 2007). The motivation to develop the BCM algorithm already very early on (Bienenstock et al., 1982) was exactly to take into consideration the “history” of a postsynaptic neuron. It is the notion, that synaptic input changes the characteristics of a synapse, and that the status of the postsynaptic neuron at any given time is a result of the inputs of its past. The term θ m, introduced in the BCM algorithm, entails a sliding threshold of synaptic modification that potentiates the synaptic weight, if the output frequency of the neuron exceeds θ m, and depresses the weight, when the frequency is below θ m. That means that LTD is induced by increased θ m in the first case, and LTP is induced when θ m is reduced in the latter case. This measure avoids to make the neuron unstable.

LTP has been investigated especially in pyramidal cells of the hippocampus, which attributes a crucial part of associative learning in a specific context to this brain region. Pyramidal neurons spontaneously generate EEG activity (Ventriglia, 2008). Small pyramidal neurons in layer 2–3 of the cortex communicate with high beta or gamma frequencies, whereas layer 5 pyramidal neurons rather produce alpha and theta frequencies. A link between the theta rhythm and mechanisms of attention has been postulated (Vinogradova, 2001) because rhythmic oscillations in the theta (4–15 Hz), but also in the gamma (20–80 Hz) bandwiths are among the most prominent patterns of activity in attentional tasks (Buzsáki and Chrobak, 1995). Apparently, both rhythms encode essential aspects of hippocampal functions, as are learning and memory. Morphologically, they can be distinguished in local processes that are preferably associated with high-frequency oscillations in the gamma band, while long-range interactions rather synchronize at the lower frequencies of theta, alpha (8–12 Hz), and beta (13–30 Hz) bands (von Stein and Sarnthein, 2000; Siegel et al., 2012). Interestingly, networks oscillating at different frequencies can interact with each other by cross-frequency coupling (Figure 1) or phase-amplitude coupling. As a result, the power of gamma oscillations can be influenced by the phases of theta or alpha band oscillations (Canolty et al., 2006). Interestingly, recent data obtained from slices of neocortex also suggest interactions between spontaneously occurring delta rhythms in NonREM-sleep and single action potentials at theta frequency (Carracedo et al., 2013). It is believed, that these stages of sleep support information processing and memory consolidation.

Most models are built on a learning rule that is able to infer the best or most likely weights, approaches broadly consistent with analogous events found in biology, such as LTP and LTD.

However, one important element known from chaos theory appeared to be missing in this learning rule, the notion of uncertainty, which is the main strength of a probabilistic approach described recently (Pouget et al., 2013). In contrast to use a point estimate of the weights, computing a posterior distribution over weights, or averaging over the weights would be more robust. In this context, the authors discuss models that entail rules for learning compositions of structures (structural learning) and suggest to start modeling with simple graphs and let the networks grow with increasing amounts of incoming observations and, hence, increasing complexity. In order to contain the sizes of the graphs and avoid that they reach limitations, a new set of neurons was used when a new node appeared. Although this measure may work well, it does not reflect the biology of the brain, where the appearance of new neurons is scarce. Another option would be to assume rewiring of neuronal networks with changing tasks. This also is unlikely, because the brain does not have the ability to rewire itself in a task-dependent manner. As a consequence, the structural learning process has to work with a more or less constant number of neurons and their synaptic plasticities.

Moreover, there exists another biological problem that needs to be considered in computer simulations: nonsynaptic or extrasynaptic events produced by moderate levels of synaptic plasticity (no LTP) (Saar and Barkai, 2003), adding a synergistic factor to synaptic events (Xu et al., 2005; Triesch, 2007). In the presence of long-term synaptic plasticity including LTP, robust levels of nonsynaptic activities could be generated acting as negative-feedback mechanisms to ensure network stability (Armano et al., 2000). These nonsynaptic activities can be localized in different compartments within the same neuron down to the level of single dendritic branches (Losonczy et al., 2008), extending the biological options to modulate neuronal network activities (Mozzachiodi and Byrne, 2010). In keeping with this, spiking events in dendrites have also been reported in response to action potentials generated in the soma/axon region of a neuron (“backpropagation”) both *in vitro* (Stuart and Sakmann, 1994) and *in vivo* (Spencer and Kandel, 1961; Lee et al., 2006), which affected the induction of LTP (Letzkus et al., 2006). This stimulated the search for voltage-gated ion channels in dendrites. Sodium channels facilitating backpropagation have been identified in pyramidal neurons and may induce local action potentials (Losonczy and Magee, 2006). Furthermore, voltage-gated Ca2+ channels of the L-, T-, and R-types and of the P/Q- and N-type (Markram et al., 1995; Kavalali et al., 1997; Bloodgood and Sabatini, 2007) have been studied in dendrites of various neuronal cell types. Spike backpropagation, local spike initiation and synaptic potentials likely are regulated by K+ channels (Hoffman et al., 1997; Bekkers, 2000), a variety of which are expressed in dendrites. Additionally, voltage-dependent Cl^−^-channels (Madison et al., 1986) and hyperpolarization-activated (h) channels (Pape, 1996; Lörincz et al., 2002; Kole et al., 2006) have been detected. Apparently, the spatial density gradient of h channels along the dendrites displays resonance frequency maps, where inputs into different regions could become differentially filtered (Narayanan and Johnston, 2007). This filtering function can result in fine-tuning of neuronal oscillatory behavior. Gating of all these channels is highly dependent on their cellular environment and subject to regulation by phosphorylation/dephosphorylation both of which can occur within milliseconds but can also be stable for extended periods of time (Johnston and Narayanan, 2008). Moreoever, induction of synaptic plasticity could also be elicited by one trial of a conditioning, tonic depolarization of the resting potential, and followed by a second trial. Theoretically, these activities can be envisioned as many local, dendritic networks functioning along with some long-distance projections that together are reminiscent of so-called small-world networks first described mathematically by Watts and Strogatz (1998). Viewed from the systems level, the network of dendritic ion channels and their plasticity could be conceptualized alongside with synaptic plasticity. The integration of all these aspects of real biological systems into future models of learning theory and computational neuroscience requires quite some work in the years to come (London and Häusser, 2005; Kim and Linden, 2007; Tretter et al., 2010; Tretter and Gebicke-Haerter, 2012).

Along these lines, but distinct from those dendritic networks, specific dendritic networks connected by gap junctions have to be discussed (Bennett and Zukin, 2004; Connors and Long, 2004; Hormuzdi et al., 2004). These specific contacts, so-called electrical synapses, formed between neuronal dendrites are channels constructed by connexins (Frisch et al., 2005), encompassing important ways of cell-cell communication. They can be viewed as syncycial neuronal networks. A hemichannel inserted in one cell makes contact with a hemichannel of a neighboring cell, in this way not only allowing flux of ions, but also of additional small molecules from one cell to another. Hence, small synaptic proteins can enter neighboring synapses and mediate cross-talk between dendritic spines. Synaptic proteins are indeed shared between neighboring synapses (Gray et al., 2006). These dendritic networks are believed to be part of a synaptic engram (Tsuriel et al., 2006). Apparently, there are five different connexins (Cx26, Cx30.2, Cx31.1, Cx36, and Cx45), expressed in neurons of the brain (Venance et al., 2004; Vandecasteele et al., 2006; Kreuzberg et al., 2008). Connexin36 is the major neuronal connexin (Condorelli et al., 1998) expressed by GABAergic, fast-spiking, parvalbumin-positive neurons throughout the mammalian brain. These neurons form gap junctions between their dendrites, or between their dendrites and somata (Fukuda et al., 2006). Moreover, gap junctions have been observed also in excitatory pyramidal neurons (Fukuda, 2007). This manner of cell-cell communication permits a much faster transfer of information than the well-known neurotransmission between pre- and postsynaptic domains. Gap junctional intercellular transmission has been found to be either bidirectional or rectifying (Phelan et al., 2008). However, gap junctional proteins and resultant cellular networks are not only expressed and maintained by neurons, but—equally important—expressed by glial cells, as discussed and extended below. There are even reports of gap junctional connections between glial cells and neurons (Alvarez-Maubecin et al., 2000). Activities through these networks could be reconciled with two models of clustered or dispersed plasticity in processes of learning and memory developed some years ago, that involve dendritic cross-talk following LTP or LTD either in random synapses of the dendritic arbor or concentrated in nearby synapses. In these models, the formation of an engram could be understood as the result of processes of enhanced synaptic protein synthesis in potentiated synapses connected by dendro-dendritic arborisations (Yuste and Urban, 2004; Govindarajan et al., 2006).

Obviously, these investigations on neuronal networks understand them as devices accepting and storing information in digital form, because of the well-known fact, that a neuron fires or does not fire. The brain, however, is not only composed of neurons. There are now numerous reports attributing a major function of information processing and storage to glial cells, to astrocytes, in particular. These cells, along with oligodendrocytes, are in intricate connection with neurons at their synapses and their axons, and astrocytes conduct electrical currents preferably on shorter distances and in more graded ways reminiscent of analogous processing elements. This is owed to the fact that the density of K+ channels in astrocytes by far exceeds that of Na+ channels, preventing the generation of glial action potentials. Therefore, it is attractive to presume that the brain exploits both the digital and analog ways of information processing and storage.

### Glial fine-tuning of neuronal electric activities

The contribution of glial cells to the formation and maintenance of engrams has been largely ignored in the past. This important issue has only been discovered very recently, giving rise to a workshop held at the National Science Foundation in Arlington, Virginia, which assembled an international team of experts on learning and memory together with experts on glia (Fields et al., 2014). In contrast to the view held decades ago, that astrocytes are mere supportive elements of neuronal networks in the CNS (glia = glue), they are now recognized as important cells closely communicating with neurons and modifying their activities (Araque et al., 2001). As outlined below, astrocytes appear to be an important cellular interface to control and modify neuronal data processing and flow of information by their close physical contact with neuronal fibers (Clarke and Barres, 2013). During development of the CNS, they serve functions of guidance to migrating neurons and subsequently help in the elaboration of synaptic contacts by refinement and specialization of their fibers (Ullian et al., 2001). Apparently, this facilitation of synapse formation by contact of astrocytes with neurons is mediated by integrin-dependent signaling pathways (Hama et al., 2004). It raises the possibility that astrocytic networks with specific temporal and spatial characteristics of information processing closely interact with distinct information processing of neuronal networks. These features have been implemented in some simplified computational models of artificial neural networks to study the effectiveness of long term synaptic facilitation (Wallace and Bluff, 1995).

**Figure 2:**
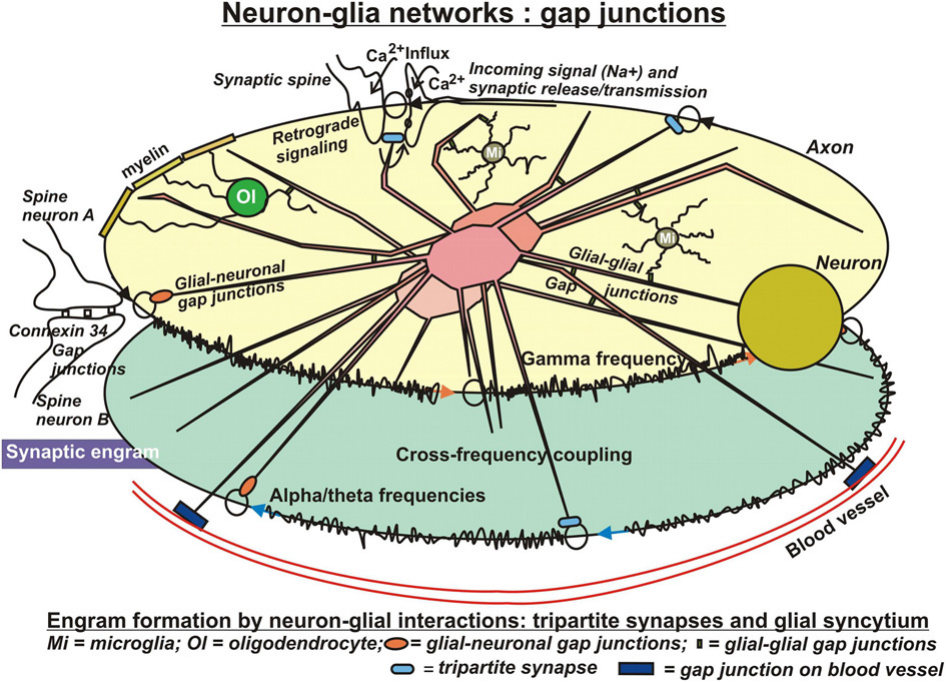
**In search for substrates of engrams**. Evidently, the neuronal networks used in computational models are insufficient to reflect the biology of learning and memory. Clearly, the glial compartment of the brain is missing. Possibly the most important cell type in this respect is the astrocyte. Astrocytes are closely connected with most, if not all neuronal synapses, form a network with each other, the astrocytic syncytium, are connected with other glial cell types and additionally with the vasculature. The tremendous number of processes of human astrocytes (approximately 20 times more than rodent astrocytes) confers to them an ideal role in distribution and long-term storage of information. In particular, in sharp contrast to neuronal networks, the salient feature of these highly complex astrocytic networks of communication is their analog processing of incoming stimuli, because these cells do not produce action potentials. This allows for graded, fine-tuning of data processing and storage. Moreover, it permits modification or even control of neuronal network activities by neurotransmitter uptake, release, and retrograde signaling events. Conversely, activities of astrocytic networks are under control of neuronal input at synaptic clefts but also through connections by gap junctions. In conclusion, engram formation in the Central Nervous System presumably proceeds and consolidates by close interactions of two distinct but mutually complementing, cellular network systems. Unfortunately, information on the roles of other glial cell types in this respect are sparse. Oligodendrocytes may have substantial influence on neuronal electric properties by their tight contacts of myelin with axons, and microglia appear to modify astrocytic activities as well as synaptic neurotransmission upon inflammatory stimuli. Additionally, microglia may influence neurotransmission under normal conditions, which gave rise to the notion of a “quad-partite” synapse.

Astrocytic fibers intimately ensheath dendritic spines (Campbell and Shatz, 1992), adapt their motility to the behavior of dendritic spines and are able to rapidly elaborate contacts with or retract from the synapse (Haber et al., 2006). In this way, astrocytes get in direct contact with the released neurotransmitters (Derouiche et al., 2002) and mutually communicate by gap-junctions (Theiss and Meller, 2002) (Figure 2). This structural peculiarity gave rise to the concept of “tripartite” synapses, encompassing the pre- and postsynaptic neurons and the astrocyte. Recent observations revealed that calcium signals from astrocytes connected to those synapses are essential for their normal function (Tanaka et al., 2013). It has been reported, that processes from a single astrocyte can envelop up to 2 million synapses in the human brain (Oberheim et al., 2006) and approximately 140,000 synapses in rodents (Bushong et al., 2002). This salient feature of human astrocytes may result in superior cognition, learning and memory typical only for humans in the light of a recent study in mice using human astrocytes transplanted in the forebrain. These animals showed improved LTP and learning abilities (Han et al., 2013). Apart from the well-known uptake system for glutamate in astrocytes by glutamate transporters at glutamatergic synapses, their communication with neurons is also mediated by astrocytic G-protein coupled receptors (GPCRs) and Gq as the G-protein responsible for astrocytic Ca2+ elevations. These receptors enable astrocytes to respond to additional neuroligands (Shelton and McCarthy, 2000; Perea and Araque, 2005) by elevations of intracellular Ca2+ (Dombeck et al., 2007; Bekar et al., 2008) and enable this cell type for information processing and storage (Perea and Araque, 2006). In the absence of neuronal stimuli, most astrocytes in hippocampus, cortex, and thalamus also exhibit spontaneous Ca2+ oscillations (Aguado et al., 2002; Nett et al., 2002). These oscillations are synchronized upon rises of neuronal activities in neuronal networks (Aguado et al., 2002). As a matter of fact, astrocytes respond to synaptic activity by local discrete calcium transients that occur on a similar time scale to that of synaptic activity (Panatier et al., 2011). In 1994, Haydon et al. (Parpura et al., 1994) made the seminal observation in cultured astrocytes, that increased intracellular Ca2+ concentrations ([Ca2+]i) induce release of glutamate. This finding has been confirmed lateron *in vivo*. This in turn implies that the release of gliotransmitters might lead to the synchronization of neuronal firing patterns (Fiacco and McCarthy, 2004). Indeed, astrocytes activated by neurotransmitters may in turn release glutamate into the synaptic cleft and activate postsynaptic neuronal glutamatergic receptors (Angulo et al., 2004; Fellin et al., 2004; Jourdain et al., 2007). Hence, astrocytic Ca2+-elevations transiently can increase the synaptic efficacy. Along these lines, it has been shown that astrocytic release of glutamate synchronously activated neighboring neurons through extrasynaptic NMDA receptors in CA1 of the hippocampus (Angulo et al., 2004; Fellin et al., 2004). These authors showed that the synchronization by glial cells was activated by neuronal activity itself, which suggests the existence of a reciprocal neuron–glia regulatory feedback loop. It is important to note, that this synchronous activation was spatially restricted to neurons with less than 100μM separation, which would assume a regional glial network that conveys a spatially limited degree of synchronization. In addition to glutamate, astrocytes have been shown also to release D-serine, an unusual amino acid produced from L-serine by serine racemase. It has been reported recently, that the release of this amino acid by astrocytes in the hippocampus was crucial for the induction of NMDA-dependent LTP (Henneberger et al., 2010), challenging for the first time the neuro-centric concept of LTP. It is a confirmation of earlier reports on direct stimulation of single astrocytes in the hippocampus (Perea and Araque, 2007) and in the hypothalamus (Gordon et al., 2009) that resulted in prolonged potentiation of synapses (Todd et al., 2006). Optionally, astrocytic Ca2+-elevations can also enhance the probability of presynaptic release (Pr) rather than influence postsynaptic elements or extrasynaptic receptors. Indeed, it was shown that the synaptic potency was unchanged in the presence of increased Pr after astrocyte stimulation (Perea and Araque, 2007). In keeping with this, astrocytes apparently can also inhibit Pr and serve a “buffering” function on synaptic activities, the so-called “glial scaling.” In this process, that proceeds within hours to days, the firing rates of a neuron are maintained in an optimal range by scaling the strengths of all synaptic inputs to that neuron up or down to balance out the relative intersynaptic differences in efficacy. Without such compensatory scaling, an increase in synaptic efficiency would rapidly reach saturation of neuronal firing rates and disable the neuron's responses to changing stimulation patterns. Conversely, a decline in synaptic strength would reduce the neuronal probability to pass the threshold for triggering an action potential and, in consequence, silence the neuron (Fregnac, 1998; Feldman, 2002). Collectively, those data suggest that only few astrocytes are involved in local, homosynaptic modulation and plasticity, not requiring communication within a glial network, whereas heterosynaptic events encompass glial communication via a glial syncytium (Ben Achour and Pascual, 2010).

Such a functional syncytium with communicative behavior is composed of astrocytes coupled together by gap junctions. However, there is also some evidence for significant neuron-glia gap-junctional coupling in a few brain regions (Figure 2). Those gap junctions help to coordinate cell firing in neuronal networks and adjust metabolic and transcriptional activities between coupled neurons and astrocytes (Dere and Zlomuzica, 2012). Recently, Pannasch et al. (2011) showed that coupled astrocytes are involved in synaptic transmission at CA1 pyramidal cells. Here, astrocytic gap junctions mediate extracellular glutamate and potassium removal during synaptic activity, modulate neuronal excitability and neurotransmitter release, and participate in the insertion of postsynaptic AMPA receptors. Moreover, they can change morphology and turnover of neuronal spines through interaction of their ephrin3 with ephrin A4 (EphA4) receptors on spines (Murai et al., 2003; Slezak et al., 2006). These results suggest that astrocytic gap junctions in the hippocampus play an important role in the regulation of both synaptic transmission and plasticity and possibly memory formation (Rouach et al., 2008; Pannasch et al., 2012; Escartin and Rouach, 2013). Collectively, this would be an exciting field for computational neuroscience and modeling. Unfortunately, until now, little efforts have been undertaken to include astrocytes and other glial cell types into computational approaches.

Astrocytic gap junctions are mainly formed by connexins 43 and 30 (CX43 and CX30, respectively) in a cell type-specific fashion (Nagy and Rash, 2000). Connexins 30 are the main proteins mediating intercellular coupling between radial glial cells in the adult dentate gyrus (Kunze et al., 2009), whereas Cx43 is the main constituent of the brain-spanning astrocytic gap junction network (Yamamoto et al., 1992). Another connexin expressed in astrocytes is Cx26 (Koulakoff et al., 2008). In the human genome, 21 and in the mouse genome 20 different connexin genes have been found, coding for distinct connexin proteins (Willecke et al., 2002). A gap junction channel consists of two hemichannels (connexon) which are contributed by two neighboring cells. Each connexon is composed of six connexin proteins.

Gap junction channels formed by connexins mediate the propagation of intercellular Ca2+ waves. Moreover, through these cellular networks, astrocytes can exchange molecules, such as K+ or glutamate (Rottingen and Iversen, 2000), or permit the intercellular, bidirectional diffusion of nutrients, of other ions, metabolites, or second messengers, such as Ca2+, cAMP, IP3, and more small molecules of up to one kDa or less than 16°A in diameter (Dobrowolski and Willecke, 2009). The gating of gap junction channels in the brain is regulated dynamically (Giaume and McCarthy, 1996). They are able to vary their conductance (Yang et al., 1990), their subunit composition, the number of cell contacts and show activity-dependent plasticity. Changes of channel conductance depend on transjunctional voltage, intracellular Ca2+, on intracellular pH, on sodium and magnesium concentrations, on phosphorylation or cytokines (Dermietzel, 1998; Salameh and Dhein, 2005). For instance, phosphorylations of Cx43 by protein kinases including mitogen-associated protein kinase (Warn-Cramer et al., 1998), protein kinase C (Lampe, 1994) or tyrosine kinase (Loo et al., 1995) exhibit strong influences on the astrocytic gap junctional network. Phosphorylation of Cx43 induces the uncoupling of cells and suppresses gap junction-mediated intercellular signal transfer. Apparently, this transfer from astroglia to neurons through Cx43 hemichannels is required to consolidate fear memory (Sáez et al., 2003; Stehberg et al., 2012).

The electrical properties of gap junctions coupling hippocampal astrocytes are distinct from those of electrical synapses between neurons (Meme et al., 2009). In contrast to neurons, activation of GABAA receptors in astrocytes causes Cl^−^ efflux, which results in astrocytic membrane depolarization (Bekar and Walz, 2002). This receptor-mediated depolarization induces a rise in cytosolic [Ca2+]i (Meier et al., 2008). With higher [Cl^−^]i, GABA application can mediate bidirectional Cl^−^ fluxes in astrocytes, Cl^−^ efflux via GABAA receptors, and Cl^−^ influx along with GABA uptake via GABA transporters. GABAA receptor-mediated currents are propagated via gap junctions within the astrocytic network. The suggested mechanism: GABA spillover activates astrocytic GABAA receptors localized near the synaptic clefts, and their signals propagate to neighboring astrocytes via gap junctions. Such homeostatic dynamics of Cl^−^ within the astrocytic network might contribute to maintain efficient neuronal GABAergic transmission by regulating [Cl-]_o_ (Egawa et al., 2013). These examples convincingly show, that the (astro-) glial syncytium should not be considered as a separate cellular system, but rather as intimately intertwined with neuronal networks. In other words, astroglial wiring is influenced by neurotransmitters, or peptides released by neurons, or by cytokines and endogenous lipids, released by other brain cell types, including microglia and endothelial cells (Giaume et al., 2010). Overlaps of these networks with functional units of neurons have been shown in the somatosensory cortex (Houades et al., 2008) and the glomerular layer of the olfactory bulb (Giaume et al., 2010).

Under the view, that coupled astrocytes can communicate with each other via the propagation of calcium waves and with surrounding neurons via the release of neurotransmitters (such as glutamate), as well as through other extracellular signaling molecules (such as ATP), possibly released through connexin43 hemichannels (Kang et al., 2008; Stehberg et al., 2012), it is evident that they play a much more active role in information processing and higher cognitive functions than previously assumed (Fields and Stevens-Graham, 2002; Nedergaard et al., 2003). In keeping with this, ATP does not only serve as a source of energy supply, but also as a ligand for purinergic receptors that induce a rise of intracellular calcium to expand transglial waves in local neural circuits (Hoogland et al., 2009). This increase of intracellular calcium via P2Y receptors also controls transmitter release by induction of heterosynaptic LTD in the CA1 region of hippocampus, in this way keeping at bay ongoing LTP (Chen et al., 2013). Therefore, in addition to memory storage by neuronal networks, mutual interactions between glial and neuronal networks may increase the efficiency to organize memory, and substantially extend storage capacities.

Furthermore, there is close contact of astrocytes with blood vessels via gap junction proteins that decorate blood vessel walls (Figure 2). These astrocytes form a physical link between the vasculature and synaptic terminals. It is a network subserving a metabolic supportive function by facilitating glucose delivery from the blood to neurons. In this network, again, the astrocyte is not a passive element but metabolizes glucose into lactate and releases it to sustain neuronal synaptic activities (Pellerin et al., 2007; Pellerin and Magistretti, 2012). As reported by Suzuki et al. (2011), a transient disruption of the flow of energy substrates from astrocytes to neurons severely interferes with subsequent formation of long term memory, although it does not markedly affect learning. This complementary network supposedly is extremely important in pathological conditions such as hypoglycemia or hypoxia to ensure neuronal survival, because in those conditions gap-junctional channels are still functional (Cotrina et al., 1998). Most importantly, however, the lactate derived from glucose or glycogen is pivotal in non-pathological conditions, where it is required for learning and memory (Suzuki et al., 2011).

Finally, it has to be mentioned, that the glial network of information processing and storage not only encompasses astrocytes, but also other glial cell types. For example, oligodendrocytes form heterotypic gap junctions with astrocytes *in vivo* consisting of Cx32 and Cx47 at the oligodendrocyte and Cx30 and Cx43 at the astrocytic side, constituting part of a panglial network. And oligodendrocyte—oligodendrocyte gap junctions have been observed, as well (Nagy et al., 2011; Wasseff and Scherer, 2011). The panglial gap junctions, in particular, appear to be essential for maintenance of normal myelin (Tress et al., 2012). Moreover, even subtle disturbances of the intimate contact of oligodendroglial myelin membranes with neuronal fibers can change conduction velocities, with ensueing alterations in the oscillatory behavior of neuronal networks (Pajevic et al., 2013). In inflammatory or hypoxic conditions, this network seems to be extended to connections with microglia that are also able to communicate via gap junctions (Eugenín et al., 2012). These cells may participate in eliminating synapses (pruning), but also in facilitating new synaptic contacts under non-inflammatory conditions. Hence, normal synapses have been considered very recently as composed of four distinct cells, the pre- and postsynaptic neurons, the astrocyte and the microglial cell, named the “quad-partite” synapse (Schafer et al., 2013).

### Molecular basis for memory formation

#### Synaptic restructuring and translation

Memory is in significant part a molecular process through which learned information is stored (Klann and Sweatt, 2008). Until now, relatively little is known about the formation of molecular engrams. Research on changes on the molecular level specific for memory have predominantly focused on molecules involved in synaptic plasticity.

The synaptic tagging and capture hypothesis (Frey and Morris, 1997; Redondo and Morris, 2011) includes a functional change in synaptic strength of an activated synapse accompanied by a temporary structural remodeling of the cytoskeleton resulting in the exposition of local (dendritic) tags and involving a large number of proteins and their interactions. Simultaneously, the synthesis and distribution of plasticity-related proteins (PrPs) is upregulated. Neither the functional nor the structural changes will persist without the supply and incorporation of new PrPs. Only if these proteins are captured in some specific way by the tags, a memory trace can be consolidated. A tagged synapse that has received PrPs, will stabilize its new structural conformation before the tagging state fades and so maintain its change in synaptic efficacy. The structural remodeling of the spine including the actin network, and the role of CaMKii required for tagging, therefore, seem to be necessary but not sufficient conditions for the expression of long-term (L)-LTP (Lang et al., 2004; Redondo et al., 2010). There is an initial increase in the number of AMPA receptors inserted into the available post synaptic density (PSD) slots of existing dendritic spines. In the case of early (E)-LTP, the number of release sites and AMPA receptors gradually return to baseline levels. By contrast, for L-LTP, the supply of PrPs anchors the additional AMPA receptors via new PSD slots, that are matched by a sustained and complementary increase in release sites. Eventually, remodeling of spine structure results in an increase (L-LTP) or decrease (L-LTD) in the number of slots available for AMPA receptors, and a corresponding presynaptic change in vesicle release sites (Lisman and Raghavachari, 2006).

Possibly subtle, local changes in the rates of synthesis of a variety of proteins are sufficient for memory consolidation. This leads us to another notion raised some years ago, that only a small but functionally effective localized, quantitative change in specific protein translation is necessary to induce transient, short-term memory that is then stabilized by positive feedback mechanisms requiring only minimal ongoing constitutive protein synthesis (Bailey et al., 1989; Kelleher et al., 2004). It is assumed that these events occur locally at a dendritic spine, and that the process is NMDA receptor-dependent. The primary site of translational control is the initiation step of binding the mRNA to the small 40S ribosomal subunit and its positioning to the initiation codon. A decline of translation initiation occurs upon phosphorylation of the α subunit of the initiation factor eIF2 at Ser51, which prolongs its association with eIF2B and consequently inhibits GDP/GTP-exchange (Sonenberg and Dever, 2003). There is some evidence, that phosphorylation of this factor influences L-LTP and contextual fear conditioning (Costa-Mattioli and Sonenberg, 2006). Another factor investigated in this respect is mechanistic target of rapamcyin (mTOR) and its effector molecules (Graber et al., 2013a,b). It has been reported recently that memory consolidation during sleep is mediated by the mTOR pathway (Seibt and Frank, 2012).

#### Epigenetics and transcription

**Figure 3:**
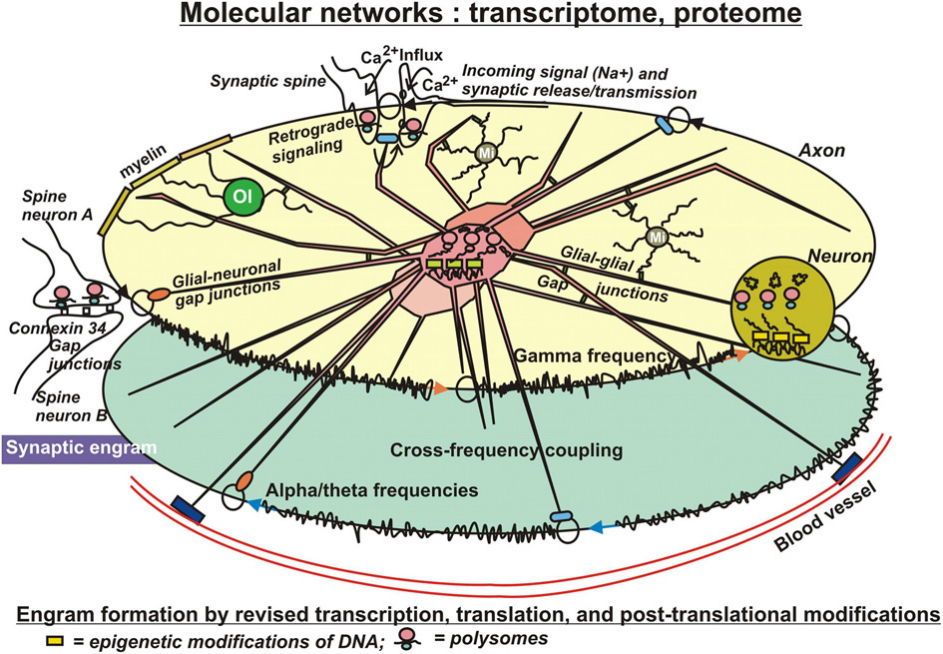
**In search for substrates of engrams**. Eventually, patches of memory, or engrams have to be laid down in structural details of molecules. Significant modifications on that level include transcriptional, translational, and post-translational adaptations to environmental impact. Adverse external influences can result in changes of DNA and histone modifications (epigenetics), with consequent changes of transcription, in altered translation, and in a wealth of readjustments of secondary or tertiary modifications of protein structures, actions that can even occur in pre- or postsynaptical sites, where ample polysomes have been identified, prepared for fast responses to new challenges. These subtle structural modifications of molecules appear to be best suited as physical entities for engram formation. Their covalent bonds ensure stability for extended periods of time, but nevertheless entail the option to be reversible. Interactions of those “memory” molecules within their molecular networks introduce long-term, but slow adaptations with far-reaching long-term consequences, such as altered ion channel conductances, altered synaptic mobility or release properties, or modified astrocytic lactate supply of neurons under hypoxic stress. The sustained, dynamic interactions of the organism with its environment further imply additional exposures of these molecular mechanisms to both favorable and adverse influences, which can result in compensation of prior adverse modifications or in reinforcement of existing adverse modifications by more modifications. This notion conforms with the concept of schizophrenia and other mental illness to develop by accumulation of repeated insults afflicted by negative, environmental conditions.

Very unlikely, changes on the genome level have substantial influence on the formation of engrams, because the relative stability of the genome does not allow for the required, rapid adaptations to environmental challenges. Therefore, factors controlling transcription are amongst the first to consider in the present context. The available literature strongly supports the notion, that altered transcription is a necessary component at least for the longest-lasting forms of synaptic plasticity and memory (Figure 3). The “classical” way to regulate transcription is by the action and interactions of transcription factors. In the light of epigenetic influences, their binding to respective, specific responsive elements on DNA is, at least in part, dependent of those prior DNA modifications. Nevertheless, quite some transcription factors have been identified to be required for short- and long-term memory, as well. These include several immediate early gene products, such as the transcription factor CCAAT enhancer binding proteins (C/EBPs), c-Fos, and Zif268, as well as effector gene products such as activity-regulated cytoskeletal protein (Arc) and tissue-plasminogen activator (TPA) (Melchor and Strickland, 2005; Alberini, 2009). In nucleus accumbens, overexpression of the transcription factor CREB increases overall excitability of neurons by enhancing the Na+ current while suppressing the K+ current (Dong et al., 2006). Furthermore, CaMKiiβ could act on a pool of actin to mediate spine expansion (Sanabria et al., 2009). This polymerization of F-actin has been visualized via live imaging within single dendritic spines (Ahmed et al., 2006). Brain derived neurotropic factor (BDNF) is another factor inducing a whole array of genes. At least part of them are believed to be associated with learning and memory (Liao et al., 2007). Evidently, these approaches are rather searching for influences of single gene products, but are not driven by a systems biological concept trying to find molecular networks working in concert to shape and consolidate long-lasting memory.

Important control mechanisms for transcriptional rates are modifications of DNA (methylations) and of histone proteins, commonly subsumed under epigenetics. Many of those modifications are introduced in response to environmental influences and can be considered as fine-tuning mechanisms of gene expression. They can produce short- and long-lasting, quantitative changes in gene expression that might serve as mechanisms to erase or consolidate traces of memory. Accordingly, a number of recent studies have focused on the role of epigenetic changes in memory formation and storage (for review, Jarome and Lubin, 2013; Zovkic et al., 2013). Epigenetic control of transcription serves as a means to maintain increased or decreased levels of specific proteins associated with engram formation at the synapse or in other cellular compartments. Changes in methylation have been identified as a possible maintenance mechanism of memory, partially because these changes are relatively stable over time. DNA methyl-transferase (DNMT) 1, for example, appears to actively maintain at least some existing methylation patterns (Law and Jacobsen, 2010; Zovkic et al., 2013). It does not mean, however, that methylations are not reversible. An example of the fine-tuned repression/derepression of a gene by methylation has been described by Miller and Sweatt (2007). The suppression of memory traces has been observed in association with the activity of protein phosphatase 1 (PP1). One result of PP1 activity is the methylation of the promoter of the Reelin gene. The Reelin protein reportedly is involved in long-term memory formation (Weeber et al., 2002). Upon methylation of the PP1 promoter by DNMT1, normal memory is restored along with demethylation of the Reelin promoter by an as yet ill-defined demethylase. However, as mentioned in the Introduction, short-term memories may be transferred from the hippocampus to the cortex for long-term storage. Correspondingly, it has been shown, that in early memory tasks methylation of the Reelin promoter is rapidly reduced in hippocampus, but reconstituted to normal within 24 h. By contrast, reduced promoter methylation persists for extended periods of time in areas of the prefrontal cortex (Miller and Sweatt, 2007; Sui et al., 2012). This example is mentioned to indicate that kinetics, duration, and readout of DNA methylations and demethylations vary in different brain regions, cell types, and genes, and may be important molecular traces of wide-spread distribution of long-term memory in the brain. Therefore, investigations on genome-wide methylation patterns in different brain regions at different times after a learning task could reveal changes in transcriptional networks guiding to gene products involved in engram formation. It is currently unclear whether other epigenetic mechanisms, such as histone acetylations and nucleosome remodeling are involved in memory maintenance. There is ample evidence, however, that changes of histone methylation occur in concert with DNA methylation in the adult CNS. Histone lysine methylation, for example, which can either activate or repress gene expression depending on the number of methyl groups associated with a specific lysine residue, has been shown to be dynamically regulated following context fear conditioning (Gupta et al., 2010). In this behavioral learning paradigm, long-term down-regulations of histone H3 lysine 4 trimethylation (H3K4me3) have been observed in the entorhinal cortex 24 h after the learning task (Gupta-Agarwal et al., 2012). It would be interesting to identify all the genes that are affected by these trimethylation changes in these conditions and construct a transcriptional network. The observation that histone modifications are subject to faster turnover than DNA methylations is reminiscent of the considerable dynamics of this molecular system, that is additionally paired with a tremendous complexity contained in the innumerable combinations that are possible to tag histone tails. More details of histone modifications are discussed below, because they belong to issues of post-translational modifications (PTMs). These types of epigenetic changes appear to encode short-term rather than long-term memory formation. But, apparently, we are far away from understanding the multiple subtle alterations in the molecular patterns of the astounding network of the epigenetic code. A more thorough update of these mechanisms is being provided in an accompanying paper (Sananbenesi and Fischer, in review).

Finally, it has to be noted, that transcription of mRNAs does not necessarily need to be confined to the neuronal cell body. In 1982, Steward and Levy reported on the occurrence of polyribosomes in dendrites and spines of hippocampal pyramidal neurons, meaning that mRNAs are protected from degradation by binding to ribosomes in the cell body and transported on microtubules to postsynaptic sites of dendrites or to axon terminals (Figure 3), where the above mentioned plasticity-related proteins (PrPs) could be synthesized directly on demand. Using microarray technology, Eberwine et al. (2002) and Matsumoto et al. (2007) have identified hundreds of mRNAs in neuronal dendrites, confirming the possibility of local protein translation (Martin and Zukin, 2006). Those polysomes provide the synapse with a fast mechanism to translate mRNAs in that specific compartment of the neuron into functional proteins in response to neuronal activities (Graber et al., 2013a,b). Microarray analyses of transcripts and of translated proteins on genome-wide levels likely hold promising potential to identify molecular substrates of engrams, in particular, if they are conducted and combined on temporary scales.

#### Post-translational mechanisms

Consolidation of short-term memory probably requires protein synthesis and PTMs, as well (Figure 3). PTMs have been studied in a wide range of proteins including the histones. These proteins can be Lys acetylated, mono-, di-, or trimethylated, biotinylated, ubiquitinylated, NEDDylated, SUMOylated; Arg methylated; Ser/Thr/Tyr phosphorylated; and Glu ADP ribosylated all occurring within 50–100 residues on the N-terminal and C-terminal tails of H2A, H2B, H3, and H4 (Bhaumik et al., 2007; Latham and Dent, 2007), which is additionally paired with a tremendous complexity contained in the innumerable combinations that are possible through these modifications (Histone Code). Histone phosphorylations and their interactions have been studied extensively. They can be considered as logic gates (Lim, 2002) consisting from “write,” “read,” and “erase” PTMs (Lim and Pawson, 2010), where the kinases are the writers, phosphatases are erasers and protein-protein interactions are mediated by modular domains (e.g., SH2 domains) that bind to a tyrosine- or serine-/threonine-containing linear motif, in this manner reading the phosphate tags. There are large numbers of phosphate transferases (>500 protein kinases) and large numbers of hydrolases (>140 protein phosphatases). Very often, histone tails are phosphorylated at more than one amino acid. Theoretically, if the two sites are phosphorylated by different protein kinases, this configuration could provide a logical AND gate in a downstream response. In general, protein kinases exhibit quite strong selectivity for the primary sequence around the residues that they phosphorylate. Therefore, the presence of multiple PTMs and the new binding motifs that they form could result in lower specificity requirements for writer domains. If this is a general principle, it provides an important mechanism for decoupling catalytic activity from specificity in proteins: catalytic domains could focus solely on their catalytic function, while other domains would specifically bind to the substrate of the catalytic reaction. Furthermore, phosphorylation can prevent or promote Lys acetylation or methylation and vice versa. Another example is acetylation and methylation of histine H3K9, which are mutually exclusive, providing an example of direct competition. The histone H3 N-terminal tail can be simultaneously methylated at K4 and acetylated at five different lysines (K9, K14, K18, K23, and K27) (Taverna et al., 2007). Furthermore, methylation and ubiquitination can stimulate Lys acetylation, and SUMOylation antagonizes histone acetylation. There are more indirect effects of phosphorylations on other modifications. Phosphorylation of H3S10 prevents recognition of HP1 chromodomain of H3K9m3e; conversely, a chromodomain bound to H3K9me3 precludes phosphorylation of S10. Hence, in this example one protein connects different PTMs on two histone tails (Ruthenburg et al., 2007). Most importantly, these examples imply a dynamic component. Post-translational protein phosphorylations have also been studied specifically focusing on the role of PKMζ (Pastalkova et al., 2006; Kelly et al., 2007). PKMζ overexpression has been demonstrated to enhance memory (Shema et al., 2011), suggesting that PKMζ indeed plays a role in memory stability (Kwapis and Helmstetter, 2013). PKMζ is known to increase AMPA receptor trafficking to the synapse to actively maintain potentiation (Yao et al., 2008). Furthermore, following a learning event, the cytoskeleton is rearranged to establish strengthened connections in activated synapses, including changes in AMPA receptor expression at the postsynaptic density. In this context, other protein kinases are discussed as well. For example, PKCλ/ι has been shown to be responsible for AMPA receptor phosphorylation and synaptic incorporation during LTP (Ren et al., 2013). Together, these studies suggest that increases in PKMζ and cytoskeletal rearrangement work in concert to maintain LTP. “Writer,” “reader,” and “eraser” functions, as described above for histones have been assigned to the components of many other PTMs in non-histone proteins (Seet et al., 2006). For instance, it has been shown that two or more phosphorylation sites in a protein can have a combinatorial effect on activity. Proteins primed through phosphorylation by one protein kinase are often phosphorylated processively on the N-terminal side of the priming phosphate by GSK3 at a series of Ser/Thr spaced by three residues, with the cluster of phosphates regulating protein activity (e.g., glycogen synthase, beta-catenin). There is also extensive use of acetylation, methylation, phosphorylation and other PTMs in nonhistone proteins, resulting in positive or negative regulation. For example, positive regulation would result through acetylation of a protein that depends on whether this protein has been phosphorylated (primed, see above), hydroxylated or ubiquitinated beforehand. An example of negative regulation (a PTM competing with phosphorylation) would be the attachment of O-linked N-acetylglucosamine residues, which are coupled to specific Ser/Thr in many types of proteins, with transcription factors being prominent (Hart et al., 2007). Negative crosstalk of any sort between different PTMs can in principle be used as an OR logic gate in a signaling network. Unfortunately, very often it is not known whether different sites on an individual protein molecule are simultaneously modified. Consequently, determinations of all PTMs coexisting on a single molecule—and their changes over time are warranted.

The notion that engrams are formed and stored in defined neuronal cell populations as more or less permanent entities after a consolidation process resulting in increased synaptic strength (Liu et al., 2012), has been challenged vigorously since a long time (Routtenberg, 2013). As proposed more than 40 years ago (Routtenberg, 1972), information storage and retrieval emerges from a reciprocal, rapidly oscillating interaction among competing processes to permit the semblance of co-occurrence. In consequence, the hypothesis was put forward, that stable memories do not require stable synapses. Stabilization of synapses would even be contraproductive, because it prevents the physiological malleability of brain circuitry that is essential for proper memory retrieval. Stabilization of synapses, therefore, interferes with the construction of long-lasting memory. In other words, there is no long-term storage of memory, no dual traces, but rather a multiple representation of prior events by dynamic, self-reinforcing but impermanent networks (Routtenberg, 2008a,b). This notion has been supported by convergent findings some time ago using resolution imaging of dendritic spines that reveal dynamic, ongoing structural flexibility measured in seconds (Matus et al., 2000). The migratory habits of synaptic proteins indicate that the molecular composition of synapses is in flux, and the molecules themselves are subject to (secondary/tertiary) modifications in a sustained updating according to environmental input. Synapses, hence, are not stabilized by LTP, as posited in the long-standing concept, described above, but undergo sustained reshaping morphologically and very likely also in their functions depending on the incoming stimuli. From these data and the PTM hypothesis developed in parallel, it can be concluded, that the central Hebb dogma that cells that “fire together, wire together” is very unlikely to hold for establishing long-term memory. Instead there are mutually self-regulating molecular modifications and molecular networks on pre- and postsynaptic sites that are rapidly oscillating. The status of the PTMs is dependent on the activity of the network. To this end, synapses and engrams are “stabilized” by regulated feedback mediated by the circuit in which the synapse is embedded. This ensures that at any given time new learning information can be inserted into existing networks by subtle changes of PTMs. Furthermore, if we extend our view to the involvement of glial cells and their molecular repertoire, PTMs in glial cells should also play important roles in memory formation. Phosphorylations and ubiquitinations are amongst the most studied PTMs in gap junction proteins. For example, phosphorylation of Cx43 turned out to be extremely complex and, hence, can be used for fine-tuning of channel properties. It includes at least 21 different phosphorylation sites and a growing list of more than 10 different kinases and phosphatases. But also SUMOylations, nitrosylations, hydroxylations, acetylations, methylations, and γ-carboxyglutamations, regulating their open probability, conductance and selectivity, have also been identified. Although there are many *S*-nitrosylation sites in proteins, only few of them appear to be used for this modification. Notably however, because ischemia and/or hypoxia are associated with an increased production of NO, the increased hemichannel permeability of Cx43 has been associated with Cx43 *S*-nitrosylations (Retamal et al., 2006). Hence, *S*-nitrosylations play an important role in regulating hemichannel permeability in astrocytes (Retamal et al., 2009). Cx43 can also be directly acetylated, adding another option of gap junctional regulation in astrocytic networks (Axelsen et al., 2013).

In summary, this model entails mechanisms involving positive feedback, protein synthesis, and PTMs, in an integrated fashion, and it is the interplay of the three mechanisms that allows memory storage. The post-translational level, in particular, appears to be of major importance in terms of engram formation. Changes on that level encompass covalent molecular modifications, that are relatively stable over extended periods of time, but are reversible, as well. Hence, it is an ideal playground of nature to store, to select and discard, and to refresh memory for any period of time. The huge versatility of PTMs can affect conduction characteristics of sodium-, potassium-, and other ion channels, gap junctional permeabilities, hence the network properties discussed above, and properties of receptors, structural proteins, enzymes and of innumerable other molecules in subtle and specific ways, that it is feasible to identify pivotal cues of memory formation on that level. It could even be, that long-term storage of information exclusively is confined to post-translational mechanisms (Routtenberg and Rekart, 2005). As a matter of fact, the combinatorial variability of PTMs has been dubbed recently as the PTM code (Hunter, 2007; Creixell and Linding, 2012; Minguez et al., 2012, 2013). Supposedly, not everything is in the genes, but much is in the proteins.

#### The temporary aspect

In the view that consolidation of memory develops during time and memories are not absolutely stable, even on a long-term basis (Alberini, 2011), high throughput technologies used to investigate engram formation at various time points are necessary and probably the best way to eventually obtain the required insights. Undoubtedly, these approaches would require tremendous efforts considering the different time-scales of short- and long-term memory, and the different brain regions tentatively involved. Moreover, presumably there are distinct qualities of memory in experimental animals, which are the organisms of choice for those experiments, compared to human beings. A possible simplification of technological conditions may be, that no sophisticated methods are required to investigate down to single cell levels. Concluding from the above said, that molecular systems from glial cells and neurons closely interact in memory formation, analyses of molecular networks of cellular ensembles supposedly reveal better insights in their specific interactions.

In summary, the search for molecular substrates of engrams is still in its infancy, owing to the greater complexity of molecular systems compared to electrical circuitries of neurons or neuron-glial hybrid networks. Until now, approaches are mostly hypothesis-driven and focused on single candidate molecules located in synapses. It is clear, however, that engrams are composed of molecular networks, changed and modified by environmental factors, that continue to influence each other over time and are additionally modified by new networks growing from sustained environmental impact—processes maintained during the whole life of an organism. The identification of such molecular ensembles is the challenge of the years to come.

## Brain pathology and engram formation

After having revisited the existing knowledge from literature about different ways and different levels of construction, consolidation, and long-term storage of memory, we want to relate these insights to tentative mechanisms leading to chronic psychiatric disorders with focus on schizophrenia. One of the most popular hypotheses of schizophrenia is the neurodevelopmental hypothesis that posits an induction of the disorder during pregnancy or around birth. Nevertheless, it takes many years until a reliable diagnosis can be made. The progression of the disorder goes unnoticed until early adulthood, which entails a slow but steady accumulation of subtle, adverse events during development of the brain. Clearly, these adverse (environmental) influences are distinct between affected individuals and, therefore, result in formation of patterns of engrams characteristic for the disorder in their sum, but distinct in each patient. Consequently, despite common traits, histories and outcomes of schizophrenia are individually distinct. This entails the tacit agreement, that a genetic predisposition is of minor importance compared to environmental impact. The predominant influence of environmental factors is also widely accepted for other mental disorders, like major depression and Alzheimer's disease. For those reasons, it is prudent to suggest that research efforts should focus on the mechanisms described here, beginning at the level of epigenetics, and projects solely focusing on alterations of the DNA sequence (SNP, CNV etc.) should be considered with caution. For obvious reasons, outcomes of the latter approaches have proved to be disappointing. In general, epidemiological studies are confounded by complex cause and effect relationships, unclear mechanisms by which non-shared environmental factors mediate disease risk, and an *inability to reconcile the “heritable” component embedded within what appears to be an environmental domain* (Petronis, 2010). First, the temporary or dynamic aspect is completely missing. Second, if some of the genetic variants reached significance levels, the magnitude of the effect of these variants in altering risk, as measured by odds ratios, is generally modest, in the 1.1–2.0 range. One example of such investigations is the meta analysis on the A1166C variant of the angiotensin II type 1 receptor (AGTR1) and its association to coronary heart disease (CHD), encompassing 53 studies including 20,435 cases and 23,674 controls and covering a total study time of 15 years. The authors produced odds ratio for CHD of 1.10 (95% CI 1.03–1.19), which completely disappeared when the analysis was restricted to the 11 larger studies with at least 500 cases in each (*OR* = 0.992, 95% CI 0.944–1.042) or to the 8 studies which were of high quality (*OR* = 0.990, 95% CI 0.915–1.072) (Kronenberg and Lamina, 2010). There are also some recent GWAS performed in schizophrenia patients using SNPs to search for single gene defects. The ISC data set included 3322 schizophrenia cases and 3587 controls and approximately 1 million SNPs were analyzed (International Schizophrenia Consortium, 2009). Another data set (SGENE) examined 314,868 SNPs in 2663 schizophrenics and 13,498 controls (Stefansson et al., 2009). In a third data set (MGS), 2681 schizophrenics and 2653 controls were investigated (Shi et al., 2009). It is accepted practice in these types of studies where high quantities of markers and high amounts of samples are included, to adjust for multiple comparisons and set a criterion for type I error at *p* < 5 × 10^−8^. In these conditions, not even one marker in any of the three separate data sets achieved this level. No significant associations were found (Wahlsten, 2012). Third, once a variant is identified to be statistically associated with a disease, it does not mean that the variant is in fact functionally responsible for altering an individual's disease susceptibility. Fourth, when the effect of multiple risk alleles is estimated using a simple additive model, apparently the combined effect does not explain why these complex disorders have such a strong familial occurrence, a conundrum referred to as the “missing heritability” problem. Fifth, the identification of an association of a gene allele or CNV with a disorder does not permit any clues as to when, how long, at which time, to what extent, in what brain regions the gene product is expressed, and in what molecular or cellular networks it is embedded. In conclusion, the DNA is only the hardware or the blueprint, the rest is self-organization within the limits of the software continuously modified by the environment. In other words, as stated more provocatively by D. Noble: “DNA is not the sole transmitter of inheritance,” “There is no genetic program,” (a term invented by Jacob and Monod), and “There are no other programs at any other level” (Noble, 2013). In consequence, it is very unlikely that complex mental disorders can be retraced to a set of genes with some sets of specific mutations.

For the great majority of mental illness (admittedly, there are few exceptions) it is well accepted, that they can be characterized as so-called “spectrum disorders,” disorders with smooth transitions from discreet to more severe forms or partially overlapping with other disorders. Eventually, each disorder is individually distinct and is dependent on the development of the affected individuals over time, just to repeat the extreme importance of the temporary aspect already mentioned above. For instance, there are reports on relatives of schizophrenic families, who clearly have high similarities of genetic background and showed signs of schizotypy (mild forms with no clinical deficits), but who never came down with diagnosis of schizophrenia (Chapman et al., 1994). These results can be explained by the above said, that despite inherited, “adverse” genetic repertoire, there is sufficient room available to contain these preconditions by favorable environmental conditions.

The accumulated evidence above permits to draw several important conclusions for schizophrenia research: it is trivial to say that gene transcription is distinct in cells of different organs with more or less 50% of all genes silenced (by epigenetic mechanisms) to encode and maintain cell type specificity. Moreover, in an organ like the brain, transcription in one cell or a group of cells of the same cell type may be different depending on their localization. Well-known examples are the pyramidal neurons of the hippocampus mentioned above. Their communication, that is restricted to shorter distances within the high gamma frequencies and contrasts with frequencies in the theta or beta ranges used for long-range communications, supposedly stems from distinct ion channel properties expressed by neurons located in different layers and, hence, in different environments of the hippocampus. Along these lines, schizophrenia has been associated with abnormal amplitude and synchrony of oscillatory activity of neuronal networks, in particular, at high (beta/gamma) frequencies, with evidence for impaired beta/gamma-band oscillations (Uhlhaas and Singer, 2013). Impaired task performance during a perceptual organization task was accompanied by a widespread deficit in the power of gamma-band oscillations between 60 and 120 Hz. Not only the amplitude but also the synchronicity of gamma oscillations was reduced in schizophrenia patients. Accordingly, reduced long-range phase synchronization could lead to a functional disconnection syndrome to eventually constitute a core impairment in schizophrenia. Evidence for an involvement of disturbed beta band oscillations in cognitive deficits in schizophrenia was reported by Ford and Mathalon (2008). Therefore, further research into neural oscillations should also take into account the possibility that the impairments in high frequency oscillations are related to alterations in low-frequency bands, in particular in the theta and alpha frequency ranges, and study more closely the cross-frequency coupling (Figure 1).

But also astrocytes supposedly display differential expression patterns depending on their locations in brain regions, on their cellular contacts (perivascular, perisynaptic, and contact with other glial cell types), in glia-neuronal/glial-glial networks and resulting specific cell-cell interactions. As pointed out above, astrocytes can communicate by expressing receptors or uptake transporters for neurotransmitters, by releasing signaling molecules for neuronal receptors, and by various forms of gap junctions between astrocytes and neurons and only between astrocytes. For instance, in tripartite synapses nonfunctional astrocytic receptors may cause an unconstrained synaptic information flux. Moreover, supposing that in the astrocytic syncytium gap junctions normally form plaques that function as memory devices, loss of function of gap junctions may also cause cognitive impairment (Mitterauer, 2011). The gap junction protein Cx43 is mainly expressed in astrocytes. However, under pathological conditions, it is upregulated in microglia (Eugenín et al., 2001). In both astrocytes and microglia, Cx43 can form functional hemichannels and gap junction channels (Orellana et al., 2011), which play a fundamental role in physiological and pathological processes, and participate in the inflammatory responses of glial cells (Bennett et al., 2012). This introduces an additional crucial system of the body to be considered in terms of learning and memory. There is quite some evidence, that a well-balanced communication between the nervous system and the immune system ensures normal functioning of the organism. Therefore, disturbances in normal function of the immune system lead to impairments in cognition and in neurogenesis (Marin and Kipnis, 2013), which indicates, that engram formation is influenced by the well- or malfunctiong of peripheral systems, as well. Interestingly, increased expression of immune system-associated proteins in brain has been interpreted as indication of ongoing chronic autoimmune processes, which may be true for multiple sclerosis, or for chronic inflammatory processes, like the ones observed in Alzheimer's dementia. However, in the case of schizophrenia there is no compelling evidence of an auto-immune basis. Some studies find upregulated, some others downregulated inflammatory genes. For those somewhat confusing data, the idea has recently been entertained, that the MHC, a gene locus repeatedly identified in GWAS of schizophrenia patients, is not indicative of an immune-related disorder, but may play a role in synaptic plasticity and brain development through nonimmune functions (McGuffin and Power, 2013). Actually, this aspect is not new. Already some years ago, it has been reported, that immunity-associated processes are functionally linked to adaptive neuronal responses, like learning and memory (Havik et al., 2007). Lateron, our microarray study in brains of schizophrenia patients revealed a large number of immune-related genes, as well, being down-regulated in superior prefrontal cortex (Schmitt et al., 2011). In this report, it has also been emphasized, supported by data accumulated from the literature, that many immune-related genes subserve distinct, but important functions in normal neurotransmission. Consequently, reduced expression of those genes could affect synaptic activities and result in compromised functions of neuronal and glial networks. Therefore, transcription, ensueing translation and PTMs in each cell or cellular compartments, like pre- and postsynaptic sites or astrocytic end feet in contact with neurons or with the vasculature, are permanently subject to processes of adaptation to micro- and macro-environmental requirements.

These issues are of paramount importance but extremely difficult to pursue on a systems level. The crucial task to pursue is to study the dynamic formation of disease-specific engrams long time before the disorder can be diagnosed. In this context, effects of repetitive mild or medium insults, experimentally inflicted, on engram formation may help to learn more about this crucial encoding of disease provoking molecular processes. To this end, many hands have to be joined to understand the underlying molecular events in more clarity.

## Summary and conclusion

In summary and conclusion, engram formation appears to begin very early on in life and very likely on several distinct levels of communication, such as on the level of electrical (digital) communication within neuronal networks (bifurcation mode of decision making), possibly as short-term memory, extending on glial networks encompassing primarily analog data processing, and finally on producing imprints (long-term memory) on the molecular levels of DNA (epigenome), RNA (transcriptome), and the proteome. These levels of communication closely interact and steadily reshape their structures like a patchwork of accumulating, countless numbers of engrams that reflect the impact of environmental influences on the organism and, hence, result in dynamic quilt-like patterns specific and unique to each individual. Assemblies of those engrams are supposed to synergize in the development of health or disease, by combinations that are critically dependent on environmental conditions. This view is in stark contrast to the wide-spread deterministic thinking, attributing a major role to genetic variations as cause for the eventual outcome. Admittedly, there are clearly disorders with high genetic background (Down's syndrome, cystic fibrosis etc.) but origins of the vast majority of psychiatric disorders will be identified on the highly dynamic levels described here. Therefore, future research should concentrate on identification of disease-specific (molecular) engrams accumulating over time and their interactions during the latent phases of disease development.

### Conflict of interest statement

The author declares that the research was conducted in the absence of any commercial or financial relationships that could be construed as a potential conflict of interest.
